# Efficient metal-free strategies for polymerization of a sterically hindered ionic monomer through the application of hard confinement and high pressure†

**DOI:** 10.1039/c8ra09242g

**Published:** 2019-02-21

**Authors:** Paulina Maksym, Magdalena Tarnacka, Andrzej Dzienia, Kamila Wolnica, Mateusz Dulski, Karol Erfurt, Anna Chrobok, Andrzej Zięba, Agnieszka Brzózka, Grzegorz Sulka, Rafał Bielas, Kamil Kaminski, Marian Paluch

**Affiliations:** Institute of Physics, University of Silesia ul. 75 Pulku Piechoty 1 41-500 Chorzow Poland paulina.maksym@smcebi.edu.pl kamil.kaminski@smcebi.edu.pl +48323497610; Silesian Center of Education and Interdisciplinary Research, University of Silesia ul. 75 Pulku Piechoty 1A 41-500 Chorzow Poland; Institute of Chemistry, University of Silesia ul. Szkolna 9 40-007 Katowice Poland; Institute of Materials Science, University of Silesia ul. 75 Pulk Piechoty 1 41-500 Chorzow Poland; Department of Chemical Organic Technology and Petrochemistry, Silesian University of Technology ul. Krzywoustego 4 44-100 Gliwice Poland; Department of Organic Chemistry, School of Pharmacy with the Division of Laboratory Medicine in Sosnowiec, Medical University of Silesia in Katowice ul. Jagiellońska 4 41-200 Sosnowiec Poland; Department of Physical Chemistry and Electrochemistry, Faculty of Chemistry, Jagiellonian University ul. Gronostajowa 2 30-387 Krakow Poland; Department of Physical Chemistry and Technology of Polymers, Faculty of Chemistry, Silesian University of Technology ul. M. Strzody 9 44-100 Gliwice Poland

## Abstract

In this paper, we have studied the effect of both hard confinement (nanoporous membranes treated as nanoreactors) and high pressure (compression of system) on the progress of free-radical (FRP) and reversible addition-fragmentation chain transfer (RAFT) polymerizations of selected hardly polymerizable, sterically hindered imidazolium-based ionic monomer 1-octyl-3-vinylimidazolium bis(trifluoromethanesulfonyl)imide ([OVIM][NTf_2_]). These two innovative approaches, affecting (in a different way) the free volume of the polymerizing system, allows the reduction of the number of toxic substrates/catalysts, satisfying the requirement of green chemistry. It was found that at both conditions (high compression and confinement) the polymerizability of monomer, as well as the control over the reaction and the properties of the produced polyelectrolytes, have increased significantly. However, it should be added that there were noticeable differences between FRP carried out under confinement and at high pressures. Interestingly, by appropriate variation in thermodynamic conditions, it was possible to synthesize polymers of moderate molecular weight (*M*_n_ ∼ 58 kg mol^−1^) and relatively low dispersity (*Đ* ∼ 1.7); while for the reaction performed within AAO pores of varying diameter (*d* = 35 nm and *d* = 150 nm), macromolecules of higher *M*_n_ but slightly broader dispersity indices (*Đ* ∼ 2.2–2.7) were recovered. On the other hand, RAFT polymerization carried out under confinement and at elevated pressures yielded polymers with well-defined properties. Noteworthy is also the fact that nanopolymerization leads to polymers of comparable *M*_n_ to those obtained at high-pressure studies but at significantly shorter reaction time (*t* ∼ 2 hours). We believe that the presented data clearly demonstrated that both examined approaches (the compression and application of alumina templates, treated as nanoreactors) could be successfully used as additional driving forces to polymerize sterically hindered monomers and produce well-defined polymers in relatively short times. At the same time, it should be mentioned that both proposed polymerization methods enabled us to omit the addition of metal-based initiators/catalysts, which seem to be a crucial step towards further development of the alternative green synthesis of polyelectrolytes in the future.

## Abstract 2

The effect of hard confinement and high pressure on the progress of free-radical and reversible addition-fragmentation chain transfer polymerizations of sterically hindered 1-octyl-3-vinylimidazolium bis(trifluoromethanesulfonyl)imide ([OVIM][NTf2]) has been investigated.

## Graphical abstract

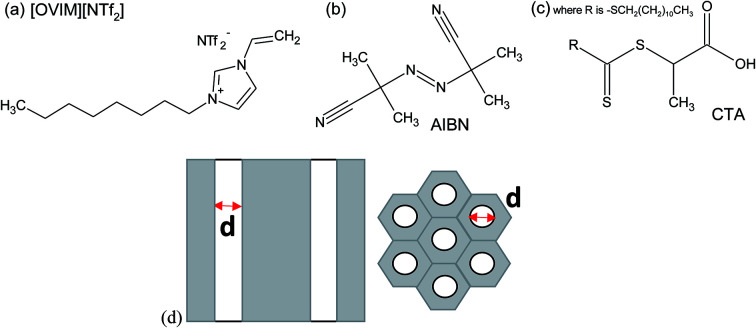

## Introduction

The synthesis of tailored macromolecules is a significant goal of the current polymer chemistry. For this purpose, many various methods have been proposed and developed. In this context, it is worth mentioning the most popular pseudo-living free-radical polymerization techniques (LRP)^1^ such as atom transfer radical polymerization (ATRP),^2^ nitroxide-mediated polymerization (NMP)^3^ or reversible addition-fragmentation chain transfer polymerization (RAFT).^4^ The critical factor in all these methods is to keep the concentration of the radicals and the number of growing chains as low as possible. As a consequence, the side reactions including termination and chain-transfer can be effectively eliminated resulting in the synthesis of polymers with a well-defined structure, topology, controlled molecular weights, narrow molecular weight distributions, and stereochemistry. However, despite the significant progress in this field, some monomers/groups of monomers cannot be polymerized or polymerization is not effective with the use of LRP due to some thermodynamic and kinetic limitations. The classic examples of that are (i) sterically hindered monomers such as α-substituted acrylates, and (ii) monomers characterized by strong specific intermolecular interactions such as H-bonds or ionic liquids, where coulombic interactions play an essential role. In this context, one can mention a quite interesting group of imidazolium-based ionic liquids (IL) with different lengths of aliphatic side chains as additional steric hindrances satisfying both criteria to become hardly polymerizable monomers. Interestingly, although the synthesis of imidazolium-based poly(ionic liquid)s (PIL)s *via* FRP has been widely described in the literature,^5–8^ previous attempts to polymerize those monomers under LRP conditions resulted in a low or moderate molecular weight polyelectrolyte with linear or block topologies (*M*_n_ lower than 200.0 kDa) and relatively high dispersities (*Đ* = 1.27–2.23).^9–11^ Moreover, polymerization effectiveness significantly dropped with the increase in the size of pendant alkyl chain as an additional steric hindrance. In fact, for the butyl or octyl moieties, the progress of polymerization was less favored, yielding low molecular weight PILs with higher dispersity. Therefore to overcome low polymerizability of these kinds of materials, where standard LRP fails, other alternative methods must be developed. Our very recent studies clearly indicated that the high pressure could be an additional driving force for the polymerization allowing effective polymerization even in the sterically hindered, strongly interacting ionic monomers. Just to remind that at high compression an increase in the conversion of ILs and better control over polymerization was achieved. Consequently, we were able to produce well-defined PILs of higher *M*_n_ up to 450.0 kg mol^−1^ and narrow dispersities (*Đ* ∼ 1.10). Our results are in line with the previous reports showing that at high pressure polymerization speeds up, side reactions are limited or well-defined stereopolymers or macromolecules of very high molecular weight and low dispersity not attainable at ambient pressure can be synthesized.^12,13^

An alternative and attractive method to synthesize polymers of tailored properties developed in recent years is the application of the geometrical constraint conditions. As widely reported in the literature, the appropriate variation in the size and functionality of constraining media allows to controlling basic properties like molecular weight, dispersity, and tacticity of the produced polymers.^14–19^ As a consequence, the confinement conditions gives us the remarkable possibility of producing materials of designed properties and defined morphology, *i.e.*, nanowires or nanotubules, dependently on the applied nanoreactors, what might be of great importance, especially for the electronic application in, *i.e.*, lithium-ion batteries,^20^ solar batteries^21^ and/or fuel cells.^22,23^ It should also be pointed out that over the years many theories have been developed to explain the role of nanoreactors in the progress of polymerization. Many of them consider a positive impact of the confined medium on the stabilization of the propagating radicals. That, in turn, leads to the suppression of the termination step and more controlled nature of the polymerization. Although, one has to emphasize that due to the strong impact of the nanoscale spatial restriction and surface interactions, on the dynamics, mobility, diffusion of the confined monomers and growing polymers, the progress of polymerization in these conditions still remains poorly understood. Additionally, one can stress that recently Kipnusu *et al.*^24^ have demonstrated that also the free volume is highly affected in nanochannels. Therefore, one should also take into account the variation in density (which seems to be lower under confinements with respect to the bulk material) on the progress of polymerization.

Since hard confinement and high pressure affect the density of the materials in a completely different way, a unique opportunity to probe the impact of free volume on the progress of free-radical and RAFT polymerization appeared. Therefore in this paper, we have studied the impact of the high pressure (high-density sample) and nanoporous templates (more free volume) on the progress of the FRP and RAFT of 1-octyl-3-vinylimidazolium bis(trifluoromethanesulfonyl)imide [OVIM][NTf_2_]. Herein, it should be mentioned that the choice of the monomer has not been accidental. Our previous investigations have shown that, independently on the counterion, with increasing size of the pendant alkyl chain polymerizability of the imidazolium-based monomer decreased significantly. For the butyl and octyl side chains, only oligomers or very low molecular weight polymers were recovered for the RAFT reactions performed at ambient pressure.^25^ Such a scenario resulted from the tendency of the reaction systems to form ordered nanostructures, where the critical length of alkyl chain for nanoorganization begins from a butyl moiety (*n* ≥ 4). At higher compression, this problem has been solved.^26^ In the current contribution, we have expanded our investigation and probe progress of FRP and RAFT of [OVIM][NTf_2_] at similar thermodynamic conditions and in AAO templates. In this context, it should be highlighted that a majority of the work published in literature is devoted to nanopolymerization of quite typical monomers such as styrene, methyl methacrylate, DGEBA-amine, urethanes, which do not provide any problems with the bulk polymerization. In contrast herein, we examined hardly polymerizable sterically hindered monomer, which is characterized by the ability to nanostructure ordering and strong coulombic interactions. Our studies clearly indicated that polymerizability and control over the polymerization had been significantly improved concerning the reaction carried out at ambient pressure yielding polymers of larger *M*_w_ and lower dispersity in much shorter times. Moreover, it was demonstrated that by appropriate manipulations of the temperature and pressure it was possible to control the progress of FRP (it mimics CRP) and obtain polymers of lower dispersities. Additionally, due to the catalytic effect of both compression and spatial restriction, it was possible to limit the number of reagents resulting in the simplification of the reaction system and eliminate toxic compounds being the part of initiators/catalysts.

## Materials and methods

### Materials

**Fig. 1:**
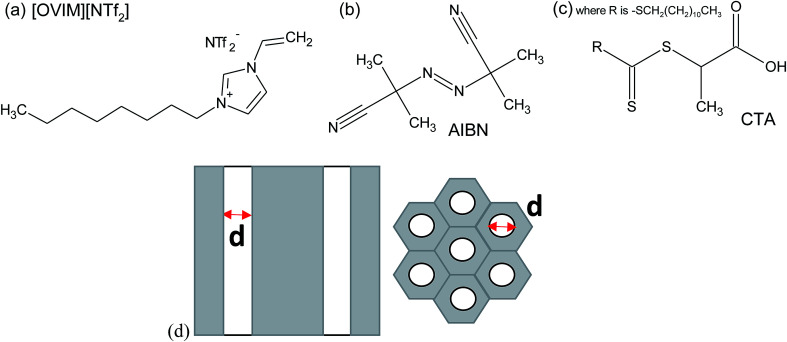
The chemical structure of the monomer (a), initiator (b) and CTA (c) and the structure of applied AAO templates (d).

2,2′-azobis(2-methylpropionitrile) (AIBN, 0.2 M solution in toluene), 2-(dodecylthiocarbonothioylthio)propionic acid (CTA), 1-vinylimidazole, iodomethane, ethyl bromide, dimethyl sulfoxide-d_6_ and LiNTf_2_ were purchased from Sigma Aldrich. The nanoporous aluminum oxide membranes of *d* = 150 nm used in this study were supplied from Synkera Co. On the other hand, AAO membranes with the pore diameter of *d* = 35 nm were prepared by two-step anodization of aluminum in stirred 0.3 M H_2_C_2_O_4_ at 25 °C.^27^ The chemical structure of investigated compounds is presented in Fig. 1 together with the scheme of applied AAO templates. Details concerning properties of AAO templates can be found in the ESI.†

### Instruments

#### Nuclear magnetic resonance spectroscopy

Proton nuclear magnetic resonance (^1^H NMR) spectra were recorded using a Bruker Ascend 600 spectrometer operating at 600 MHz in DMSO-d_6_ as a solvent. Standard experimental conditions and the standard Bruker program were used. ^1^H NMR analysis confirmed the conventional structure for all polyelectrolytes. Based on the results of NMR spectra we determined the conversion of each monomer, the degree of polymerization (DP_n_), and the number-average molecular weight of the polymer (*M*_n, th_). Note that full spectroscopic assignments, as well as the ^1^H NMR spectrum taken from the polymerization mixture, is presented in the ESI.†

#### Gel permeation chromatography

Molecular weights and dispersities were determined by gel permeation chromatography (GPC) with a Viscotek GPC Max VE 2001 and a Viscotek TDA 305 triple detection (refractometer, viscosimeter, and low angle laser light scattering). The OmniSec 5.12 was used for data processing. The apparatus was used in the triple detection mode, and absolute molecular weights (*M*_n_ and *M*_w_) and dispersities (*Đ*) obtained with calibration with a polystyrene standard. It should be pointed out that the characterization of polyelectrolytes, including imidazolium-based PILs by Gel Permeation Chromatography (GPC), is challenging owing to interactions of the ionic polymer with GPC columns. However, Matyjaszewski *et al.* proposed a universal method to characterize the molecular weight and its distribution of polyelectrolytes composed of imidazole polycations with bis(trifluoromethanesulfonyl)imide (NTf_2_) counter-anions.^28^ By adding 10 mM of LiTFSI in the THF as eluent interactions between polyelectrolyte and GPC columns could seemingly be avoided. Thus, the measurements were carried out in THF containing 10 mM LiNTf _2_ as the solvent at 35 °C and a flow rate of 1 ml min^−1^. Note that in case of GPC measurements of polymers produced under nanoconfinement, the polymer sample recovered from the AAO templates by a wash with THF was first freeze-dried under vacuum, then washed with water and again freeze-dried under vacuum. Measurements were carried out in 250 μl vial inserts with a flow rate of 0.9 ml min^−1^.

#### Differential Scanning Calorimetry

Calorimetric measurements of the isothermal reaction were carried out by Mettler-Toledo DSC apparatus equipped with a liquid nitrogen cooling accessory and an HSS8 ceramic sensor (heat flux sensor with 120 thermocouples). Temperature and enthalpy calibrations were performed by using indium and zinc standards. The sample was prepared in an open aluminum crucible (40 μl) outside the DSC apparatus. Immediately after the reaction, samples were scanned at a rate of 10 K min^−1^ over a temperature range from 140 K to room temperature.

#### Raman spectroscopy

Raman measurements were carried out using a WITec alpha300 R system equipped with a He–Ne laser (*λ* = 633 nm at 30 mW of power) and a high sensitivity back-illuminated Newton CCD camera. An air Olympus MPLAN (50×/0.76NA) objective was used. Time-series spectra were obtained in the 500–3800 cm^−1^ range by 10 scans with an integration time of 10 s and a resolution of 3 cm^−1^. Measurements were performed at *T* = 333 K using a THMS600 Linkam stage with a temperature stabilization of ±0.5 °C within intervals of 300 s. All data were manipulated by performing a baseline correction and cosmic ray removal. The spectrometer's monochromator was calibrated using the Raman scattering line of a silicon plate (520.7 cm^−1^).

### Procedures

#### Preparation of AAO templates of pore diameter *d* = 35 nm

A high-purity aluminum foil (99.999%, Goodfellow) was degreased in ethanol and electropolished at *T* = 10 °C in a stirred mixture of HClO_4_ and C_2_H_5_OH (1 : 4 vol) at 20 V for 3 min. The first anodizing step was performed at 45 V for 1 h. The resulting porous alumina layer was removed by chemical etching in a mixture of 6 wt% H_3_PO_4_ and 1.8 wt% H_2_Cr_2_O_4_ at *T* = 45 °C for 12 h. Subsequently, second anodization was performed at 45 V for 5 h. In order to fabricate through-hole AAO membranes, oxide layers were electrochemically detached in a mixture of HClO_4_ and C_2_H_5_OH (1 : 1 vol) at *T* = 0 °C by applying three anodic pulses of 60 V for 3 s each. The pulses were separated by pauses of 3 s. Finally, free-standing AAO membranes were chemically etched in 5 wt% H_3_PO_4_ at 40 °C for 5 min.^27^

#### Ionic monomers method synthesis

1-octyl-3-vinylimidazolium bis(trifluoromethanesulfonyl)imide was synthesized according to procedures described previously.^26^

#### Ambient pressure RAFT and free radical polymerizations of [OVIM][NTf_2_]

In the case of RAFT polymerization, the following mixture was prepared: [OVIM][NTf_2_] (1 g, 1.72 mmol), CTA (1.51 mg, 0.0043 mmol), AIBN (0.7 μl, 0.000344 mmol), and DMSO (1.3 ml) were placed in a flask with a magnetic stirring bar, whereas for free radical polymerization similar procedure was used for [OVIM][NTf_2_] (1 g, 1.72 mmol), AIBN (0.7 μl, 0.000344 mmol), and DMSO (1.3 ml). The polymerization was quenched after a predetermined time by cooling and exposing the reaction mixture to air. The polymer was isolated by ultrafiltration in methanol using a membrane (Millipore, Regenerated Cellulose, YM10, NMWL: 1000), and then dried under vacuum to constant mass. After ultrafiltration and drying each sample under vacuum to constant mass, polymers presented different phase depending on the molecular weight; low and moderate molecular weight PILs (*M*_n_ up to 50 kg mol^−1^) was high viscous materials, whereas high molecular weight PILs took the form of solid.

#### High-pressure RAFT and free radical polymerizations of [OVIM][NTf_2_]

All high-pressure polymerizations were carried out in 4 ml Teflon ampoules in a high-pressure microreactor purchased from UniPress. After the vial with reaction mixture was purged with nitrogen for 20 min, the flask was immersed in a Teflon ampoule. The reactor includes a hydraulic press model LCP20, and a pressure reaction vessel equipped with a temperature controller.

#### RAFT and free radical polymerizations of [OVIM][NTf_2_] under confinement

The reaction mixture was prepared with followed conditions: [OVIM][NTf_2_] (3 g, 5.16 mmol), CTA (4.53 mg, 0.013 mmol), AIBN (2.1 μl, 0.00103 mmol), and DMSO (3.9 ml). The mixture was transferred into the flask together with the AAO membrane. Note that prior to filling, AAO membranes were dried in an oven at *T* = 100 °C under vacuum to remove any volatile impurities from the nanochannels. After completing the infiltration process, the surface of AAO membrane was dried, and the excess sample on the surface removed mechanically, where the surface of the membrane was cleaned by a paper towel to moment when it was dry. In the experiment, we used membranes of different pores diameter: 150 and 35 nm. The total amount of reaction mixture incorporated into AAO membrane found to be ∼6 mg.

## Results and discussion

### High-pressure effect

As the first step of our investigation, we have performed FRP of [OVIM][NTf_2_] at ambient pressure conditions to compare polymerization rates and properties of the synthesized polyelectrolytes to those produced previously with the use of ambient pressure RAFT^25^ (see Table 1). Polymerizations were carried out in DMSO (100% v/v to monomer) with 0.5 wt% of AIBN, resulting in a very low monomer conversion reaching a maximum consumption *α* ∼12% within 48 h (IV), which is comparable to our previous results for RAFT process.^26^ However, it should be highlighted that this traditional and uncontrolled FRP did not allow to produce polymers of both high *M*_n_ and low dispersity. Herein, the synthesized polyelectrolytes were characterized by *M*_n_ up to 37.0 kg mol^−1^ and broad dispersity indices (*Đ* = 2.2–2.8). In contrast, *M*_n_ of polymers prepared by RAFT was much higher up to 49.2 kg mol^−1^ and revealed more homogeneous structures as evidenced by narrow disperisities (*Đ* = 1.3).^26^

**Table 1:** FRP of [OVIM][NTf_2_] performed at macroscale under ambient and elevated pressure

No	Pressure [MPa]	Time [h]	Conversion^a^ [%]	*M*_n_ b [kg mol^−1^]	*Đ* b
I	0.1	5	3	—	—
II		12	9	—	—
III		24	10	8.3	2.1
IV		48	12	27.1	2.7
V	500	5	30	380.4	2.4
VI		10	49	680.2/18.2	2.6/1.8
VII		24	68	1250.0/10.4	2.8/1.6
VIII		48	>99	1560.0/25.6	4.9/1.8
IX	800	5	23	9.1/2.3	2.1
X		10	48	10.2/8.2	1.9/1.6
XI		24	74	528.0/48.5	2.5/1.5
XII		48	>99	820.0/32.6	2.7/1.8
XIII	1200	5	18	38.2	1.9
XIV		24	35	58.1	1.7
XV		96	69	102.8	2.1

a Determined by ^1^H NMR, DMSO-d_6_, 600 MHz.

b Determined by GPC-LALLS, THF containing 10 mM LiNTf as the solvent, 35 °C, calculated d*n*/d*c* = 0.020. Note that the first value of *M*_n_ and *Đ* is related to the main polymeric fraction.

**Fig. 2:**
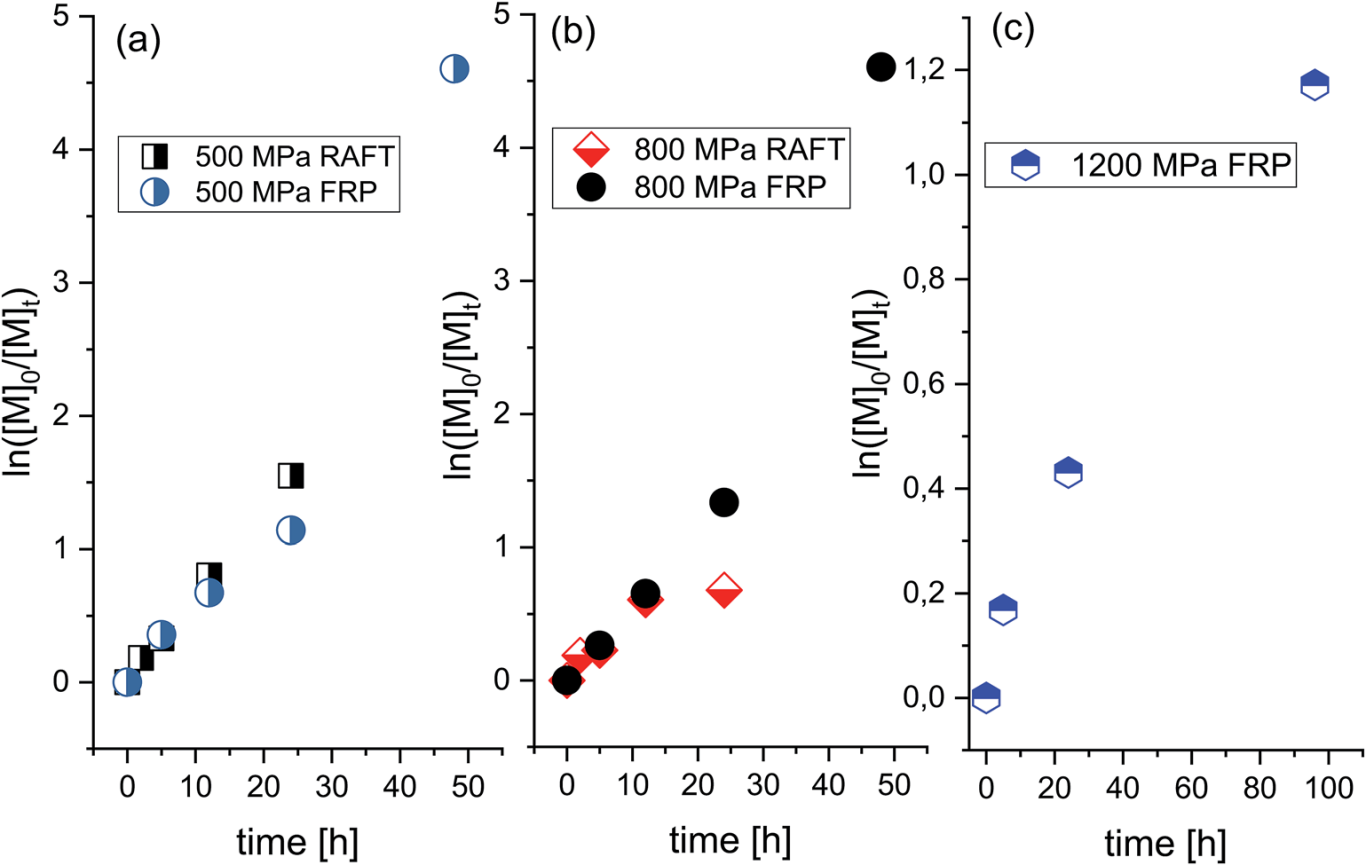
Pseudo-first-order kinetic plot *versus* conversion for RAFT and free-radical polymerization of [OVIM][NTf_2_] at *p* = 500 MPa (a), *p* = 800 MPa (b) and *p* = 1200 MPa (c).

**Fig. 3:**
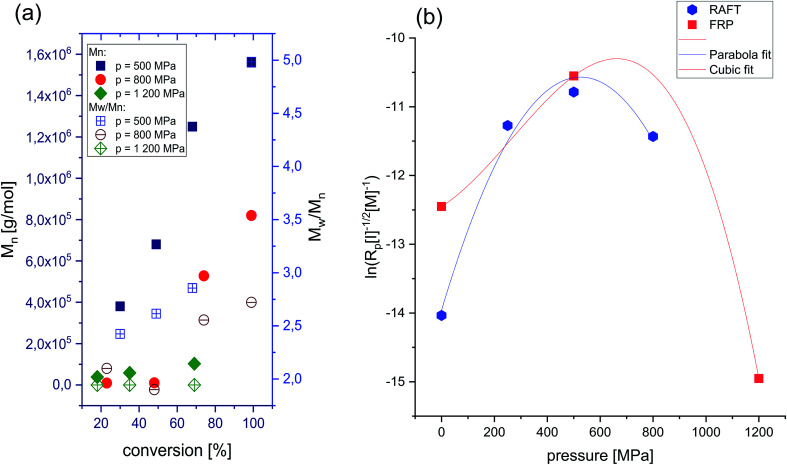
Dependence of absolute *M*_n_*vs.* conversion and *Đ vs.* conversion of P[OVIM][NTf_2_] produced at *p* = 500 MPa, *p* = 800 MPa and *p* = 1200 MPa. Note that the *M*_n_ and *Đ* values corresponding to a main polymeric fraction (a), and polymerizability factor as a function of pressure for FRP and RAFT of [OVIM][NTf_2_] (b).

In order to check whether selectivity along with the slow rate typical for the polymerization of this ionic monomer at ambient conditions can be enhanced, we decided to investigate the progress of the FRP at elevated pressure conditions (*p* = 500 MPa, 800 MPa, and 1200 MPa). It was expected that by variation in density and viscosity (due to compression), one could enhance propagation and retard the initiation and termination rates in examined systems. Interestingly, as revealed by the linear dependences of the semilogarithmic kinetic plots of ln([M]_0_/[M]_*t*_) *versus* time (see Fig. 2) and *M*_n_*versus* conversion *versus Đ* (see Fig. 3a), the “pseudo-living” character of FRP has been achieved for each of the applied high pressures: 500 MPa (V–VIII), 800 MPa (IX–XII) and 1200 MPa (XIII–XV) (see Table 1, the first values of *M*_n_ and *Đ* in Table 1 are corresponding to a main polymeric fraction, whereas the second one to an additional fraction). At *p* = 500 MPa, a value of the overall rate of polymerization (*k*) reaches *k* = 2.6107 × 10^−5^ s^−1^ for the *R*_p_[M]^−1^[I]^−1/2^ ratio calculated from the straight line, which is close to the one determined for the reaction carried out at *p* = 800 MPa (*k* = 2.65889 × 10^−5^ s^−1^). That indicates that the rate of polymerization is not or very weakly affected by a relatively strong increase in pressure (Δ*p* = 300 MPa). However, further increase in pressure by Δ*p* = 700 MPa (from 500 MPa to 1200 MPa) dramatically decreases the polymerization rate resulting in much lower *k* value (*k* = 3.20927 × 10^−7^ s^−1^, where conversion decreases from 68% (VII) to 35% (XIV) after 24 h). Note that the similar effect, *i.e.* a decrease in the reaction rate at higher compression has been observed for the RAFT polymerizations of OVIM.^26^ In that case, linear dependences of ln([M]_0_/[M]_*t*_) *versus* time plot was observed only at 500 MPa, but when pressure has increased to 800 MPa, some deviation in the linearity occurred. This scenario results from the fact that at lower pressure a critical value of the viscosity is reached at higher conversion, whereas as the pressure increases, this value moved towards the lower ones. If the system reaches a critical value of viscosity, the diffusion of the polymeric chain-ends and reversible chain transfer processes seem to proceed more slowly with respect to the propagation rate.

The influence of pressure on the polymerizability of [OVIM][NTf_2_] is shown in Fig. 3b, where the plot of the logarithm of an apparent rate of polymerization (*R*_p_ = *k*[P*][M]) is presented as a function of pressure, where [P*] is the concentration of propagating free-radicals, [M] is the monomer concentration, and *k* is the rate coefficient for propagation. In the case of RAFT and FRP processes, the logarithm of the rate of polymerization increased linearly with pressure up to 500 MPa, and then gradually decreased at the higher pressure 800 MPa for RAFT and 1200 MPa for FRP, respectively. The overall activation volumes, Δ*V*, of polymerization were calculated from:1
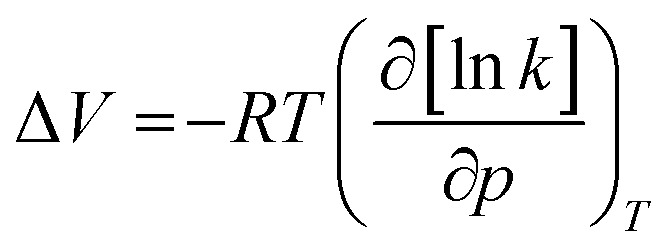
where *R* is the gas constant. From the cubic fits the pressure dependence of ln(*R*_p_) for RAFT and FRP experiments, we calculated activation volumes such as Δ*V* = −31 cm^3^ mol^−1^ and Δ*V* = −9.9 cm^3^ mol^−1^ for RAFT and FRP, respectively in the limit of low pressure (*p* = 0.1 MPa). Herein one can mention that similar values of Δ*V* were reported for the high pressure to free radical polymerization of styrene.^29^ As can be seen in Fig. 3b the impact of the compression on polymerization rate is much stronger in the case of RAFT reaction (*k* varies in the broader range). In addition, as expected the FRP process performed at ambient pressure is also characterized by a higher rate with respect to the RAFT one.

The final step of our investigation was to demonstrate the effect of high pressure on the basic properties of polyelectrolytes produced *via* FRP. It turned out that the molecular weight of the synthesized PILs decreased from 1250 kg mol^−1^ (500 MPa) to 58.1 kg mol^−1^ (1200 MPa) with pressure. Additionally, a significant drop in dispersity of the macromolecules produced at higher compression has been observed, indicating a much better control over the polymerization at more dense and viscous samples (*Đ* = 5 *versus Đ* = 1.7 for *p* = 500 MPa and *p* = 1200 MPa, respectively). An increase in pressure upon FRP allows to produce polymers of lower molecular weights but much better properties as revealed by the shape of the GPC traces which changed from nonsymmetric and bimodal characteristic for the reactions performed at *p* = 500 MPa and *p* = 800 MPa to a symmetric, and monomodal one for the polymerization carried out at *p* = 1200 MPa (see ESI†). That confirms our thesis that the termination by recombination of radicals must be significantly suppressed under these conditions. However, considering the fact that under high-pressure conditions transfer reaction are promoted, we may assume that in fact some of them have occurred. We carried out also an additional experiment for FRP at *p* = 1200 MPa to show the effect of extending the polymerization time (up to 96 h) on the progress of reaction and properties of the resulting polymer. Interestingly, for such conditions the ‘pseudo-living’ nature of polymerization was still achieved and *M*_n_ of P[OVIM] increased linearly with the conversion, reaching a value of *M*_n_ = 102.8 kg mol^−1^; however, the polymer was characterized by broader dispersity index compared to the result determined from the reaction after 24 h (*Đ* = 2.1 *versus Đ* = 1.7).

As shown above, the strong impact of pressure on polymerization of [OVIM] has been demonstrated. That includes the influence of compression on (i) polymerizability of sterically hindered ionic monomer, (ii) the rate of various steps of reaction, (iii) the basic properties of produced polyelectrolytes. In the case of the first aspect, the pressure is known to overcome to certain extent steric congestion allowing the production of polymers of higher molecular weight. This might be considered from both the thermodynamic and kinetic points of view. The first one is connected to the fact that steric congestion decreases the polymerization exothermicity and ceiling temperature *T*_c_ value due to some interactions of the rigid surroundings on the polymer backbone. Note that the *T*_c_ value is defined as the equilibrium temperature above which the polymerization becomes thermodynamically impossible. On the other hand from the kinetic point of view, the steric hindrance effect slows down the propagation step and decreases the monomer overall polymerizability. To look more deeply into this problem following aspects should be considered. First, the initiation rate is slightly reduced by compression since the application of this thermodynamic variable affects viscosity, that control diffusion of reacting chemical species as well as the kinetics of initiator decomposition. It is related to the so-called cage effect and an experimental finding demonstrating that at varying thermodynamic conditions thermal decomposition of thermoinitiators such as AIBN might be significantly retarded. In fact in ref. 30 it was clearly shown that the initiation efficiency in some solvents (*e.g.*, toluene) is independent of the temperature and decreases seemingly with pressure due to suppression of the decomposition of the initiator.^31^ Interestingly this corroborates with the general rule saying that association like reaction such as polymerization are characterized rather by negative activation volume which means that they are preferred at higher compression. On the other hand decomposition processes have negative activation volumes indicating that they are slowed down at these conditions. Therefore, the concentration of free radical in the polymerizing system at elevated pressures is most likely much lower with respect to the reaction carried out at *p* = 0.1 MPa. As a consequence of that, we can reproduce in some way conditions of controlled/‘pseudo-living’ radical polymerization (LRP) for the FRP, that is basically the uncontrolled process. In the case of propagation, pressure significantly accelerates this step with the slight increase in the rate of transfer processes. As we showed the reaction rate increases significantly up to 500 MPa, and then gradually decreases at the very high-pressure regime. Such a scenario can be attributed to the changes in viscosity and was observed for both FRP and RAFT (Fig. 3b). Note that at these thermodynamic conditions, the viscosity of the polymerized sample must be very high. However, with increasing pressure, this trend surprisingly changed dramatically. As a consequence, at higher compression progress of the RAFT is much faster. We suppose that it is an effect of both: (i) the steric congestion of [OVIM][NTf_2_] that affects both the thermodynamics and kinetics of the polymerization (competition between viscosity and *T*_c_) and (ii) differences between the mechanism of FRP and RAFT. In the case of RAFT and FRP polymerizations performed at the lowest pressures (*p* = 0.1 MPa or *p* = 250 MPa), the values of *T*_c_ and reaction temperature are probably close to each other (60 °C), indicating that depropagation also influences on the polymerizability. On the other hand, for reaction performed above 500 MPa, the propagation–depropagation equilibrium is shifted during the polymerization resulting in increasing the *T*_c_. Coming back to the chain transfer that is the reaction between a large radical and a small molecule, pressure accelerates this process, but this effect is much smaller than in the case of propagation step. This is evidenced by the constant value of molecular weights at high pressures, which results from the increased efficiency of various transfer reactions leading to the termination of the growing radical chain. Unfortunately, we observed some chain transfer processes, but the determination of which type of them provides the dominant effect (chain transfer to initiator, monomer, solvent or polymer) requires an additional experiment performed at various initiator/monomer/solvent ratios. Moreover, we suppose that in the studied herein systems the transfer to initiator process is unlikely and even it if does occurs to a very small extent. We also used DMSO as the solvent characterized by the high dielectric constant and low transfer value which might significantly decrease both the viscosity effect and chain transfer to solvent allowing to gain better control over the reaction. Finally, the termination step that is diffusion controlled process decrease at elevated pressure. In this context, at high pressure, the termination reaction has retarded due to the increase in viscosity. It should be stressed that in the polymerizing systems of very high density and viscosity an opportunity to create conditions mimicking LRP, where most likely the concentration of the propagating radicals and the rate of propagations are quite small appeared. Note that to better understanding mechanistically all the reaction steps occurring at elevated pressure more experiments including determination of the half-life of both free radical organic initiators and the propagating chain should be performed. However, it is worth mentioning that such studies are challenging to perform at elevated pressures. Taking into account the effect of high pressure on the basic properties of polyelectrolytes two main aspects should be reflected: the possibility to obtain polymers of very high *M*_n_ and possessing better properties (lower dispersity, higher functionalization) compared to those produced *via* FRP at ambient pressure. Just to mention that at most extreme conditions polyelectrolytes of pretty low dispersity around 1.7 were produced.

### Confinement effect

Next, we examined the effect of two dimensional (2D) geometrical restriction on the overall behavior of FR and RAFT polymerization of [OVIM][NTf_2_]. Both kinds of polymerizations were carried out within nanoporous alumina (AAO) membranes of different pore diameter, *d* = 150 and 35 nm. The applied constrain media as a nanoporous template made of aluminum oxide (Al_2_O_3_) composed of uniaxial channels (open from both sides) with well-defined pore size, *d* (see Fig. 1c). This matrix is an extremely stable medium, which might be reused repeatedly after the proper purification. One can note that at the moment many different constrained geometries are commonly used as nanoreactors, *i.e.*, meso and microporous zeolites, inclusion complexes, liquid and organic crystals and micelles,^32^ giving us a remarkable and easy opportunity to perform polymerization reaction that allows synthesizing materials of unique physicochemical properties, *i.e.*, morphologies controlled at the nanoscale. The progress of the FRP and RAFT nano reactions was followed by using both ^1^H NMR and Raman Spectroscopies, while the reaction products were characterized by Differential Scanning Calorimetry (DSC).

Representative Raman spectra for [OVIM][NTf_2_] monomer incorporated into the alumina membranes with *d* = 150 nm recorded at different times of the process (at the beginning and the end) were presented in Fig. 5a. It is crucial to note that [OVIM][NTf_2_] spectra recorded for RAFT and FR reactions are very similar due to the presence in the system almost the same reagents (monomer, solvent, AIBN). The only difference in chemical composition was addition trithiocarbonate-based CTA in case of the RAFT polymerization. Although, one should note that the concentration of this compound was very low. Therefore, Raman spectra were compared to each other. The subtle variations in the band position, intensity or full width at half maximum were strictly related to the structure of the growing chains of the polymer during the reaction. The change in the integral intensities, *I*, of the bands ascribed to the vibration of methylene (C=CH_2_) groups in vinyl moieties of [OVIM][NTf_2_] monomer were used to monitor the kinetics of the investigated reaction. Furthermore, a drop in the intensity of these bands with time was a clear indicator of the progress of RAFT and FR polymerization and enabled us to construct the kinetic curves for the reactions normalized according to the following equation:

2 *α*_Raman_ = (*I* − *I*_0_)/(*I*_eq_ − *I*_0_)

where *α*_Raman_ is the conversion calculated from the Raman measurements, *I* mean the integral intensity at a given time, *I*_0_ is the initial integral intensity, and *I*_eq_ is the integrated intensity in the plateau region. The analysis of the time dependence of the integral intensities of characteristic bands has indicated that polymerization proceeds upon the time and observed changes originate from the breakage of the vinyl group, which transforms into an alkane backbone s reaction proceeds. This scenario was observed in all examined membranes both for RAFT as well as FRP. One can also observe the presence of characteristic bands of the alkane backbone even in the first spectrum that most likely originates from the very fast polymerization process, which has taken place already at the moment of monomer filling or heating of the sample to the desired temperature conditions *T* = 333 K.

**Fig. 4:**
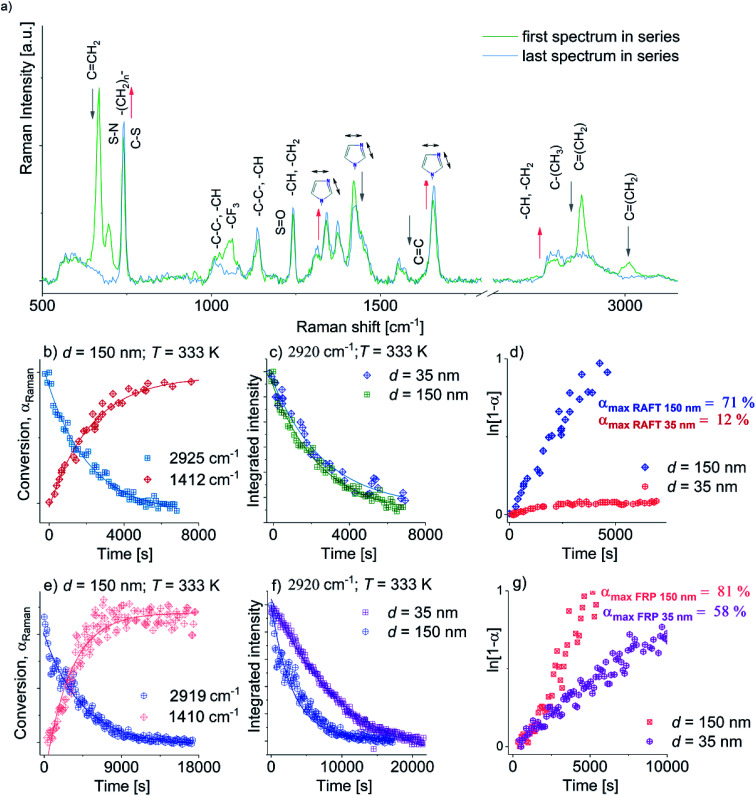
Panel (a): representative Raman spectra collected upon RAFT and FRP polymerization (spectra collected upon both polymerization reactions are without major differences) with highlighted assignments for the marker bands for the confined OVIM system measured at *T* = 333 K at the beginning (green spectrum) and at the end of reaction (blue spectrum). Grey and pink arrows indicate the decrease and increase of band intensities, respectively. Panel (b): time evaluation of *α*_Raman_ for RAFT at *T* = 333 K, *d* = 150 nm at 2925 cm^−1^ and 1412 cm^−1^. Panel (c): time evolution of the integrated intensity of the band around 2925 cm^−1^ obtained at *T* = 333 K for *d* = 35, and *d* = 150 nm; Panel (d): the plot of ln[1 − *α*] *versus* time for RAFT where *α* means the conversion rate. Panel (e): time evaluation of *α*_Raman_ for FRP at *T* = 333 K, *d* = 150 nm at 2919 cm^−1^ and 1410 cm^−1^. Panel (f): time evolution of the integrated intensity of the band 2919 cm^−1^ obtained at *T* = 333 K for *d* = 35, 150 nm; Panel (g): the plot of ln[1 − *α*] *versus* time for FRP where *α* as the conversion rate.

Next, to check the character of the FRP and RAFT polymerization under confinement, ln(1 − *α*_Raman_) was plotted *versus* time. Consequently, we obtained a series of linear dependencies that clearly demonstrated a pseudo-living nature of polymerization in both types of reactions. Furthermore, from the fitting data presented in Fig. 4d and g to the linear function, polymerization rates *k* of the FRP and RAFT polymerization carried out in pores of varying diameter were calculated. As expected, both *k* values estimated for FRP and RAFT nanopolymerizations at *d* = 150 nm were close to each other and reached *k* = 2.76945 × 10^−4^ s^−1^ and *k* = 2.14677 × 10^−4^ s^−1^, respectively. However, the crucial difference in the reaction rates between FRP and RAFT nanopolymerizations was observed for the polymerization performed in pores of *d* = 35 nm (*k* = 6.65803 × 10^−5^ s^−1^*versus k* = 1.03598 × 10^−5^ s^−1^, respectively), where the RAFT of OVIM proceeded unexpectedly slow. To explain the discrepancy between rates determined for the RAFT and FRP carried out in AAO pores, one can refer to GPC measurements that clearly demonstrated quite large dispersity of the polymers synthesized *via* FRP under confinements. In fact, GPC-LALLS analysis revealed a bimodal shape of the traces suggesting that there are at least two fractions of polymers characterized by many different molecular weights being an effect of chain transfer processes. On the other hand, the trace of the polymer produced *via* the RAFT reaction revealed symmetric and unimodal shape without any additional polymeric fractions or tailing indicating that side reactions including chain transfer are strongly reduced or suppressed in this kind of polymerization (see ESI†). Note that *k* values estimated for FRP and RAFT nanopolymerizations were almost two orders of magnitude higher compared to those calculated from the high-pressure reactions. Moreover, it is worthwhile to comment that with increasing pressure FRP polymerization become slower with respect to the RAFT. On the other hand in the case of nanopolymerization, the opposite effect was observed. Herein with decreasing pore diameter (higher degree of confinement), RAFT polymerization proceeded much slower. These effects must be related to a difference in mechanism of both kinds of polymerization. However, to find a proper explanation of this phenomenon, further studies are highly required.

The nanopolymerization conditions and properties of produced polyelectrolytes are presented in Table 2. As it can be seen, the FR polymerization performed in AAO nanochannels is significantly enhanced in comparison to the macroscale reactions carried out at ambient and elevated pressure, leading to a much higher reaction rate and shorter reaction times. The influence of the pore diameter on the polymerization rate and properties of the produced polyelectrolytes was also observed. The polymerization performed within pores of *d* = 150 nm proceeded at a faster rate compared to the other one (*d* = 35 nm), reaching 81% monomer conversion after 2 hours; whereas at the same time, the consumption of [OVIM][NTf_2_] for *d* = 35 nm was 30% lower (*α* ∼51%). In addition, the synthesized polymers were characterized by moderate *M*_n_ = 88.1 kg mol^−1^ or very high *M*_n_ = 580.2 kg mol^−1^ absolute molecular weights and high molecular weight distributions (*Đ* = 2.7 *versus Đ* = 3.1) depending on the pore diameter, *d* = 35 nm and *d* = 150 nm, respectively. Since macromolecules produced *via* FRP nanopolymerization revealed the heterogeneous structure of polymeric chains as indicated by broader dispersity indices; it is obvious that such methodology is not suitable for the preparation of well-defined polyelectrolytes. In contrast to the above results, the RAFT polymerizations proceeded in slightly slower rate, reaching a lower consumption of monomer that is 12% for *d* = 35 nm and 71% for *d* = 150 nm within 2 hours. Additionally, an unexpected very slow reaction rate for the experiment performed at *d* = 35 nm resulted in the polymer of low molecular weight *M*_n_ = 20.6 kg mol^−1^ and relatively high dispersity *Đ* = 1.39. However, the most outstanding result, concerning the application of RAFT nanopolymerization, is related to the synthesis of the well-defined polymer of *M*_n_ = 509 kg mol^−1^ (*Đ* = 1.18) by using membranes of *d* = 150 nm. Comparing the influence of pore diameter on both kinetics of RAFT and FRP and properties of yielded polymers similar dependences have been noted. Both reactions proceeded with higher rates at pores of 150 nm in respect to 35 nm, while for RAFT reaction this effect was much higher (for RAFT conversion after 2 hours increased in 59%, whereas for FRP only in 23%). In addition, polyelectrolytes obtained within pores of 35 nm are characterized by lower *M*_n_ and higher *Đ* compared to those produced at 150 nm. According to literature data, these results might be related to some changes in the local concentration of free radical at specific pore diameter. It was demonstrated that decreasing in pore diameter probably increases termination rate due to the higher value of the local concentration of radicals, which in consequence leads to a slower rate of polymerization.^33^ Considering, in turn, the impact of the spatial restriction on macromolecule properties it was evidence that the diameter of pores strongly affected on the polymer molecular weights and its distributions. The values of absolute *M*_n_ of polyelectrolytes obtained within AAO templates of pore diameter 150 nm are almost twice higher compared to theoretical *M*_n_, whereas for polymers produced at 35 nm an opposite situation is seen. Note that such correlations were observed both for FRP and RAFT. An explanation of that may be related to the production of the higher contribution of polymeric fraction situated closer to the pore walls. This effect is less important for pores of lower diameters since the interfacial layer gets much smaller. In consequence, polymers produced at lower pore diameters are much stronger physically attached to the pore walls, and therefore their extraction from the matrices is more difficult. Formation of the irreversibly adsorbed layers in thin films obtained for varied polymers supported on the aluminum substrate that confirms our results has been extensively studied by Napolitano group.^34,35^ However, to find a detailed explanation of this phenomenon, further studies are highly required. It should also be stressed that described discrepancy might also derive from the different solvation properties of poly(ionic liquid) type molecule under the chromatographic conditions used for the analysis or alternatively with the uncertainty (few percents) in the determination of the monomer conversion from the ^1^H NMR spectra.

**Table 2:** RAFT and FRP of [OVIM][NTf_2_] performed under nanoscale

No	Pore diameter [nm]	Time [h]	Conversion [%]	DP	*M*_nth_ [kg mol^−1^]	*M*_n_ [kg mol^−1^]	*Đ*
**FRP**							
1	35	2	58	—	—	88.1/12.3	3.1/2.1
2	150	2	81	—	—	580.2	2.7

**RAFT**							
3	35	2	12	47	36.5	20.6	1.4
4	150	2	71	284	221.5	509.7	1.2

**Fig. 5:**
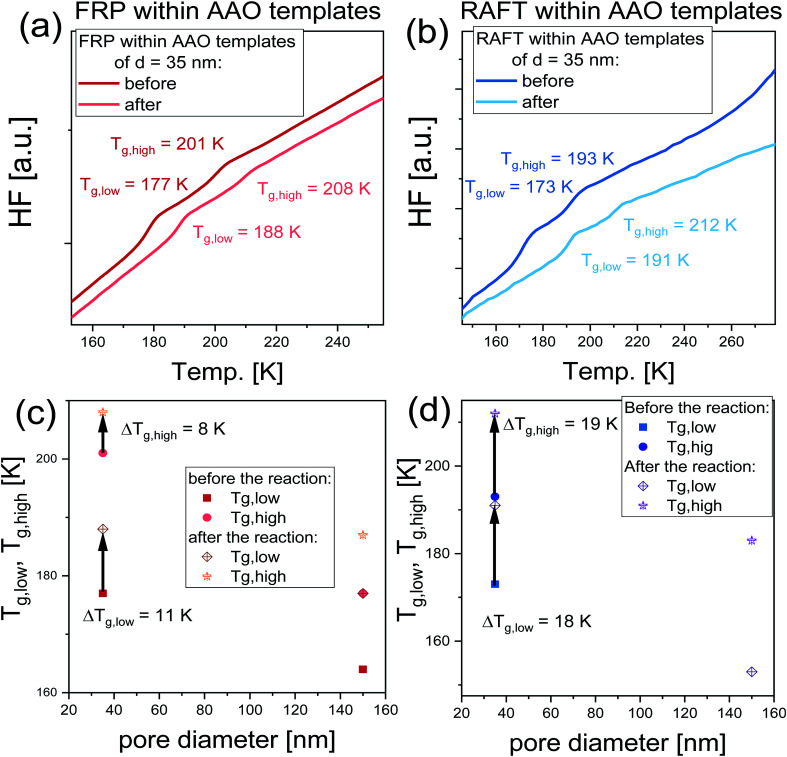
Panels (a and b): DSC thermograms collected before and after the reaction carried out within AAO templates of *d* = 35 nm; Panels (c and d): pore diameter dependence of the glass transition temperature of nanomaterials before and after the reaction.

In order to quantify the impact of the performed nanoreactions on the properties of examined nanomaterials, immediately after the reaction, the polymerized samples were measured using DSC technique, where they were cooled down to *T* = 140 K and measured with the heating rate of 10 K min^−1^. DSC thermograms collected before and after the reactions are shown in Fig. 5a and b. As illustrated, all recorded DSC signals exhibit the presence of two endothermic processes, that is a manifestation of a double glass transition phenomenon, *T*_g_. It should be mentioned that the soft matter under 2D confinement conditions exhibits double glass transitions located below and above the *T*_g_ of bulk material, widely reported in the literature for the various glass former liquids (including polymers) under 2D confinement.^36–41^ Note that accordingly to the two-layer model proposed by Park and McKenna,^42^ this double *T*_g_s phenomenon refers to the two subsets of molecules characterized by different dynamics. Thus, the following fractions can be distinguished: (i) the core molecules located in the center of the nanoporous channels characterized by the *T*_g_ lower than the bulk (*T*_g1_) and (ii) interfacial subset associated with the molecules attached at the surface of pore walls characterized by the *T*_g_ higher than the bulk (*T*_g2_). In this context, the presence of two *T*_g_s observed before the reaction corresponds to different (core and interfacial) subsets of monomer (or rather its mixture with initiator) confined within AAO templates; while, *T*_g_s detected after the reaction are related to two fractions of the nascent polymers produced at the walls of applied nanochannels and in their centers. It should be highlighted that the presence of two glass transition temperature within polymerized systems is widely reported in the literature and has been previously reported, *i.e.*, for curing of bisphenol M dicyanate ester^40,43^ and trimerization of a mixture containing mono- and difunctional cyanate ester.^44^ Values of all recorded *T*_g_s plotted as a function of pore diameter are presented in Fig. 5c and d. One can see that both *T*_g_s increase after the polymerization in each case, indicating significant changes arose within the whole examined system (at both the surface and center of the nanoporous matrix) during the polymerization. Nevertheless, it should be mentioned that this increase is higher in the case of RAFT reactions which reaches Δ*T*_g, low_ = 18 K and Δ*T*_g, high_ = 19 K; while for the FR polymerization, Δ*T*_g, low_ = 11 K and Δ*T*_g, high_ = 8 K. Note that Δ*T*_g, low_ = *T*_g, low_ (after the reaction) – *T*_g, low_ (before the reaction) and Δ*T*_g, high_ = *T*_g, high_ (after the reaction) – *T*_g, high_ (before the reaction) (the trend of changing *T*_g_s is indicated by arrows in Fig. 5c and d). We supposed that the observed Δ*T*_g_s might be a result of a difference in the monomer conversion and properties of produced macromolecules (*M*_n_ and *Đ*). It should be mentioned that in the case of FRP within AAO templates of pore diameter *d* = 35 nm, we obtained polymer characterized by high dipspersity *Đ* ∼ 2, with monomer conversion α = 51%; thus, one can indicate that the smaller increase of *T*_g_s might be due to either: (i) the presence of residual monomer, (ii) low *M*_n_ of produced macromolecules and/or (iii) significantly broad distribution of obtained polymer chains bimodal GPC signals indicate presence of at least two fractions differing in *M*_n_. Note that in the case of FPR under confinement, dispersity value reaches *Đ* ∼3 and the obtained chromatogram is bimodal, see (ESI†); therefore, one can assume that also *Đ* seems to have an important impact on the observed behavior, especially under confinement, where various polymer chains might interact with the surface differently, leading to low increase of *T*_g_. On the other hand, in the case of RAFT nanoreactions carried out in pores the produced macromolecules are characterized by *Đ* = 1.17–1.39 with a monomer conversion varying between *α* = 12–71%. Nevertheless, despite such small conversion, the difference in *T*_g_s equals Δ*T*_g, low_ = 18 K and Δ*T*_g, high_ = 19 K. Thus, one can assume that the dominant effect on the change of *T*_g_ has the properties of produced macromolecules as observed herein.

The findings reported herein clearly demonstrated that spatial restriction seems to have a stronger impact on kinetic aspects of the reaction leading to, *i.e.*, an increase of the reaction rate, a decrease of polymerization timeframe of sterically hindered [OVIM][NTf_2_]. In the context of reaction thermodynamics, one can recall that, as shown for step-growth polymerization, the reaction carried out at the nanoscale were reported to be characterized by the same activation energy as in the case of reaction performed at the macroscale.^45^ Nevertheless, it should be stressed that there is a lack of systematic studies dealing with this issue, which might be helpful in better understanding of the progress of polymerization at the nanoscale mostly because the confinement effect has not been fully understood. In this context, it should be mentioned that basic parameters such as viscosity, density, mobility, the surface tension of the soft materials incorporated into porous matrix vary significantly. Moreover, due to additional interactions between host and guest materials, there are at least two, three fractions of molecules having a completely different set of physical properties. One can also remind that wettability, contact angle, and interactions quantified by specific interactions as well as interfacial energy between porous matrix and given monomer will affect kinetics and thermodynamical parameters of the polymerizing system in porous templates.^46,47^ Moreover, one cannot forget that the soft matter under 2D geometrical constraint is characterized by higher effective free volume than macroscale material.^24,48^ In this context, the comprehensive studies carried out for various glass-forming liquids incorporated into both silica and alumina nanoporous indicate that the reduced packing density is responsible for the enhancement of their molecular dynamics.^49,50^ Therefore by the analogy, one can assume that all of these factors (finite size, surface interaction and free volume) have their own contribution and impact on the examined reaction under confinement. The observed significant acceleration of reaction time might be caused by either variation in pore diameter or free volume generated by the applied 2D hard confinement. What is more, our recent studied on the polymerization at nanoscale indicated that AIBN might decompose to free radical at 30 °C due to a specific host-AIBN interaction, which in some way caused the nonthermal activation of the applied initiator.^25^ Note that generally the application of spatial restriction affects/decreases the phase transition temperature of confined materials.^51,52^ With this in mind, we suppose that each of the effects that are the finite size, surface interaction and free value influence on the variation of both activation volume, energy and reaction enthalpy. However, as mentioned above this issue has not been addressed yet and requires further studies. In addition, nanopolymerizations performed within AAO templates were characterized by ‘pseudo-living’ nature up to the end of reaction as well as polymer molecular weights were much higher than those obtained from the polymerization performed at atmospheric pressure. Compared to the results obtained from the high-pressure studies, the confinement effect has contributed most to the reduction of the reaction time necessary to obtain a product with a comparable *M*_n_ from tens to 2 hours. However, one aspect should be pointed out, the small amount of produced polymer under confinement (a few milligrams) makes this kind of polymerization a method only for special non-commercial applications in contrast to the high pressure, cost-effective and easy industrial methodology, where polymers can be produced on a large scale. On the other hand, the controlled nanopolymerization under confinement is a method of choice to develop polymers with precisely controlled nanowires and/or nanotubules structure.

## Conclusions

As presented, we reported the first work devoted to the RAFT and FRP polymerizations of low-polymerizable, sterically hindered [OVIM][NTf_2_] carried out in two systems (high pressure *versus* confinement conditions) differing in effective free volume. Our polymerization methodology gives us the outstanding opportunity of producing polyelectrolytes with much higher *M*_n_ and lowers *Đ* within much shorter reaction time compared to the polymerization performed both at ambient pressure or macroscale. It was demonstrated that the application of hydrostatic pressure could both increase the propagation rate of OVIM and decrease the rate of initiation and free-radical bimolecular termination. Interestingly at *p* = 1200 MPa, the uncontrolled free-radical polymerization proceed in a controlled manner similar as for LRP methods yielding polyelectrolytes of moderate *M*_n_ = 58.7 kg mol^−1^ and relatively narrow disperisties *Đ* = 1.7. In the case of FRP and RAFT nanopolymerizations the using of AAO templates as nanoreactors resulted in higher reaction rates and production of polymers of better properties compared to those obtained at macroscale high-pressure conditions. The strong impact of pore diameter of molecular weights and dispersities of resulted polymers has also been demonstrated. Although it must be stressed that the ‘pseudo-living’ nature of the reaction has been reported in both types of confinement polymerizations (FRP and RAFT), in case of the former method, the content of side reactions was much higher with respect to the LRP process, resulting in polymers of broader molecular weights distributions. Additionally, in the case of nanopolymerization the applied AAO templates also enabled us to produce polymers of nanowires and/or nanotubules architecture/structure. At the same time, both proposed alternatives allowed carried out reaction without metal-based initiators/catalysts, satisfying the requirement of green chemistry, which seem to be crucial in any further advanced industrial application.

## Conflicts of interest

The authors declare no competing financial interests.

## Supplementary Material

RA-009-C8RA09242G-s001

## References

[cit1] Braunecker W. A., Matyjaszewski K. (2007). Prog. Polym. Sci..

[cit2] Controlled Radical Polymerization, ed. K. Matyjaszewski, American Chemical Society, Washington, DC, 1998, vol. 685

[cit3] Burguière C., Dourges M. A., Charleux B., Vairon J. P. (1999). Macromolecules.

[cit4] Chiefari J., Mayadunne R. T. A., Moad C. L., Moad G., Rizzardo E., Postma A., Skidmore M. A., Thang S. H. (2003). Macromolecules.

[cit5] Green M. D., Long T. E. (2009). Polym. Rev..

[cit6] Anderson E. B., Long T. E. (2010). Polymer.

[cit7] Yuan J., Antonietti M. (2011). Polymer.

[cit8] Green O., Grubjesic S., Lee S., Firestone M. A. (2009). Polym. Rev..

[cit9] Liu C., Wang S., Zhou H., Gao C., Zhang W. (2016). J. Polym. Sci., Part A: Polym. Chem..

[cit10] Zhang B., Yan X., Alcouffe P., Charlot A., Fleury E., Bernard J. (2015). ACS Macro Lett..

[cit11] Mori H., Yahagi M., Endo T. (2009). Macromolecules.

[cit12] Mueller L., Jakubowski W., Matyjaszewski K., Pietrasik J., Kwiatkowski P., Chaladaj W., Jurczak J. (2011). Eur. Polym. J..

[cit13] Kwiatkowski P., Jurczak J., Pietrasik J., Jakubowski W., Mueller L., Matyjaszewski K. (2008). Macromolecules.

[cit14] Li X., King T. A., Pallikari-Viras F. (1994). J. Non-Cryst. Solids.

[cit15] Pallikari-Viras F., Li X., King T. A. (1996). J. Sol-Gel Sci. Technol..

[cit16] Kalogeras I. M., Neagu E. R. (2004). Eur. Phys. J. E.

[cit17] Uemura T., Ono Y., Kitagawa K., Kitagawa S. (2008). Macromolecules.

[cit18] Giussi J. M., Blaszczyk-Lezak I., Cortizo M. S., Mijangos C. (2013). Polymer.

[cit19] Uemura T., Kitagawa K., Horike S., Kawamura T., Kitagawa S., Mizuno M., Endo K. (2005). Chem. Commun..

[cit20] Park M., Zhang X., Chung M., Less G. B., Sastry A. M. (2010). J. Power Sources.

[cit21] Wang P., Zakeeruddin S. M., Exnar I., Grätzel M. (2002). Chem. Commun..

[cit22] De Souza R. F., Padilha J. C., Gonçalves R. S., Dupont J. (2003). Electrochem. Commun..

[cit23] Sun J., MacFarlane D. R., Forsyth M. (2003). Electrochim. Acta.

[cit24] Kipnusu W. K., Elsayed M., Kossack W., Pawlus S., Adrjanowicz K., Tress M., Mapesa E. U., Krause-Rehberg R., Kaminski K., Kremer F. (2015). J. Phys. Chem. Lett..

[cit25] Maksym P., Tarnacka M., Wolnica K., Dzienia A., Erfurt K., Chrobok A., Zięba A., Bielas R., Kaminski K., Paluch M. (2018). Polym. Chem..

[cit26] Maksym P., Tarnacka M., Dzienia A., Erfurt K., Brzęczek-Szafran A., Chrobok A., Zięba A., Kaminski K., Paluch M. (2018). Polymer.

[cit27] Sulka G. D., Brzózka A., Zaraska L., Jaskuła M. (2010). Electrochim. Acta.

[cit28] He H., Zhong M., Adzima B., Luebke D., Nulwala H., Matyjaszewski K. (2013). J. Am. Chem. Soc..

[cit29] Arita T., Buback M., Janssen O., Vana P. (2004). Macromol. Rapid Commun..

[cit30] Zhulin V. M. (1980). Rev. Phys. Chem. Jpn..

[cit31] High-Pressure Chemistry and Physics of Polymers, ed. A. L. Kovarskii, CRC Press, Boca Raton, 1994

[cit32] Tajima K., Aida T. (2000). Chem. Commun..

[cit33] Salsamendi M., Ballard N., Sanz B., Asua J. M., Mijangos C. (2015). RSC Adv..

[cit34] Napolitano S., Sferrazza M. (2017). Adv. Colloid Interface Sci..

[cit35] Napolitano S., Wübbenhorst M. (2011). Nat. Commun..

[cit36] Jackson C. L., McKenna G. B. (1996). Chem. Mater..

[cit37] Pissis P., Daoukaki-Diamanti D., Apekis L., Christodoulides C. (1994). J. Phys.: Condens. Matter.

[cit38] Merino E. G., Neves P. D., Fonseca I. M., Danéde F., Idrissi A., Dias C. J., Dionísio M., Correia N. T. (2013). J. Phys. Chem. C.

[cit39] Golovoy A., Mazich K. A., Cheung M. F., Berry V. K. (1989). Polym. Bull..

[cit40] Li Q., Simon S. L. (2009). Macromolecules.

[cit41] Li L., Chen J., Deng W., Zhang C., Sha Y., Cheng Z., Xue G., Zhou D. (2015). J. Phys. Chem. B.

[cit42] Park J.-Y., McKenna G. (2000). Phys. Rev. B.

[cit43] Li Q., Simon S. L. (2008). Macromolecules.

[cit44] Lopez E., Simon S. L. (2015). Macromolecules.

[cit45] Tarnacka M., Dulski M., Starzonek S., Adrjanowicz K., Mapesa E. U., Kaminski K., Paluch M. (2015). Polymer.

[cit46] Alexandris S., Papadopoulos P., Sakellariou G., Steinhart M., Butt H. J., Floudas G. (2016). Macromolecules.

[cit47] Talik A., Tarnacka M., Grudzka-Flak I., Maksym P., Geppert-Rybczynska M., Wolnica K., Kaminska E., Kaminski K., Paluch M. (2018). Macromolecules.

[cit48] Tarnacka M., Kipnusu W. K., Kaminska E., Pawlus S., Kaminski K., Paluch M. (2016). Phys. Chem. Chem. Phys..

[cit49] Adrjanowicz K., Kaminski K., Koperwas K., Paluch M. (2015). Phys. Rev. Lett..

[cit50] Adrjanowicz K., Kaminski K., Tarnacka M., Szklarz G., Paluch M. (2017). J. Phys. Chem. Lett..

[cit51] Alcoutlabi M., McKenna G. B. (2005). J. Phys.: Condens. Matter.

[cit52] Alba-Simionesco C., Coasne B., Dosseh G., Dudziak G., Gubbins K. E., Radhakrishnan R., Sliwinska-Bartkowiak M. (2006). J. Phys.: Condens. Matter.

