# Supplementary material for: Efficient metal-free strategies for polymerization of a sterically hindered ionic monomer through the application of hard confinement and high pressure
Source: RSC Adv. 2019 Feb 21;9(11):6396–408. doi: 10.1039/c8ra09242g (PMC9060961; doi:10.1039/c8ra09242g)
Supplement: RA-009-C8RA09242G-s001 [file RA-009-C8RA09242G-s001.pdf]

Electronic Supplementary Material (ESI) for RSC Advances.
This journal is © The Royal Society of Chemistry 2019

Efficient metal-free strategies of polymerization of sterically hindered ionic monomer through the application of hard confinement and high pressure

Paulina Maksym[1,2*], Magdalena Tarnacka[1,2], Andrzej Dzienia[2,3], Kamila Wolnica[1,2], Mateusz Dulski[2,4], Karol Erfurt[5], Anna Chrobok[5], Andrzej Zięba[6], Agnieszka Brzózka[7], Grzegorz Sulka[7], Rafał Bielas[8], Kamil Kaminski[1,2*], Marian Paluch[1,2]

[1] *Institute of Physics, University of Silesia, ul. 75 Pulku Piechoty 1, 41-500 Chorzow, Poland*

[2] *Silesian Center of Education and Interdisciplinary Research, University of Silesia, ul. 75 Pulku Piechoty 1A, 41-500 Chorzow, Poland*

[3]*Institute of Chemistry, University of Silesia, ul. Szkolna 9, 40-007 Katowice, Poland*

[4] *Institute of Materials Science, University of Silesia, ul. 75 Pulk Piechoty 1, 41-500 Chorzow, Poland*

[5] *Department of Chemical Organic Technology and Petrochemistry, Silesian University of Technology, ul. Krzywoustego 4, 44-100 Gliwice, Poland*

[6] *School of Pharmacy with the Division of Laboratory Medicine in Sosnowiec, Medical University of Silesia in Katowice, ul. Jagiellońska 4, 41-200 Sosnowiec, Poland*

[7] *Department of Physical Chemistry and Electrochemistry, Jagiellonian University, ul. Gronostajowa 2, 30-387 Krakow, Poland*

[8] *Department of Physical Chemistry and Technology of Polymers, Faculty of Chemistry, Silesian University of Technology, ul. M. Strzody 9, 44-100 Gliwice, Poland*

**Corresponding author: e-mail* paulina.maksym@smcebi.edu.pl; kamil.kaminski@smcebi.edu.pl *phone number +48323497610*

Supplementary Materials

Properties of AAO templates

Parameter	Value	
Pore diameter [nm]	35	150
Pore density [cm^{-2}]	$6\cdot10^9$	$9\cdot10^8$
Pore period [nm]	143	367
Thickness of membranes	50µm	50µm

Table 1. Details concerning porosity, pore diameter and distribution of AAO membranes.

NMR measurements

^{1}H NMR of P[OVIM][NTf$_2$] (600 MHz, DMSO-d$_6$) δ ppm = 8.40-9.10 (m, $H_{C'}$), 7.40-7.78 (m, $H_{D'}$), 6.75-7.20 (m, $H_{E'}$), 3.60-4.50 (m, $H_{B'}$,$H_{F'}$), 1.80-2.30 (m, $H_{A'}$), 1.50-1.72 (m, $H_{G'}$), 1.10-145 (m, $H_{H'-L'}$), 0.75-0.95 (m, $H_{M'}$).

Each monomer conversion was calculated by comparing the integrations of vinyl protons of the remaining monomers (5.42 and 5.95 ppm) with the integration of methyl protons for P[OVIM][NTf$_2$] at δ=0.75-0.95 ppm.

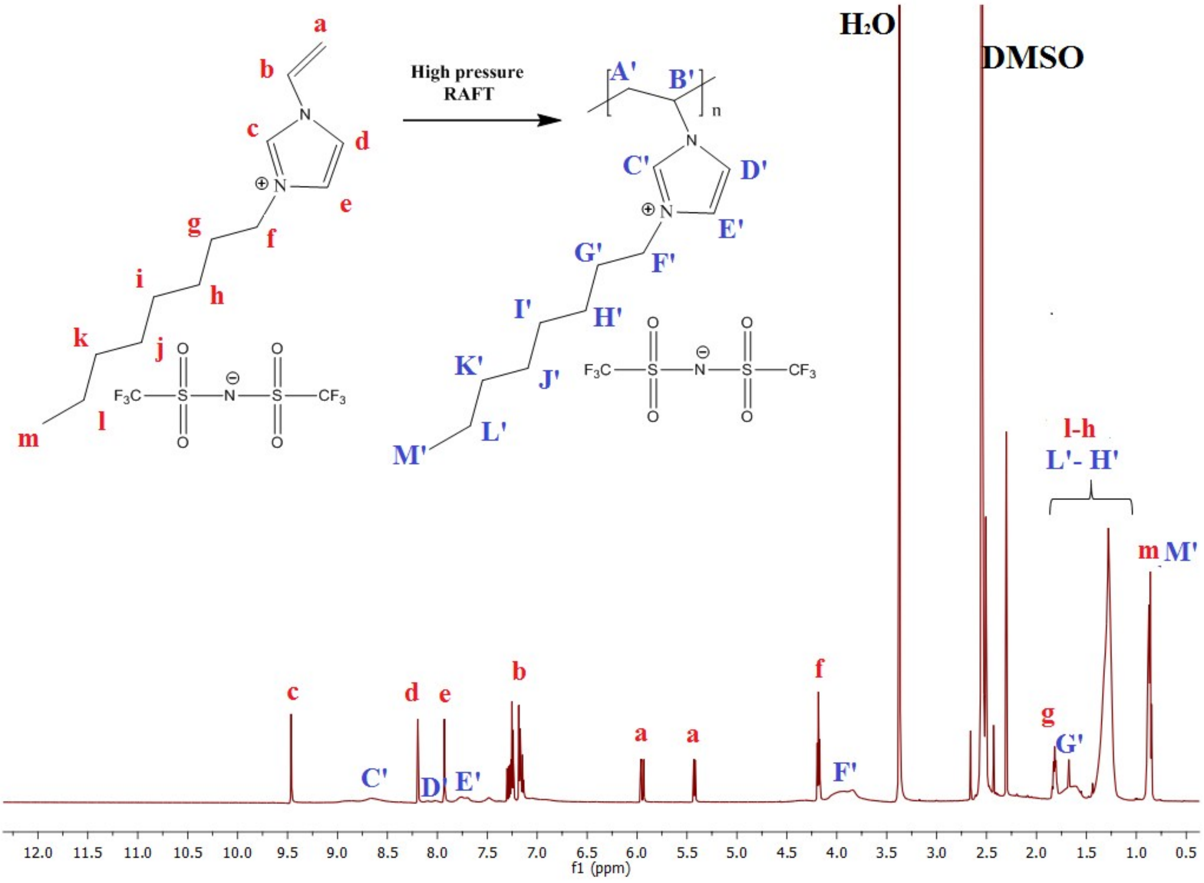

Figure 1. ^{1}H NMR spectrum of the sample taken from the reaction mixture; p=800 MPa, example of XI .

GPC-LALLS measurements

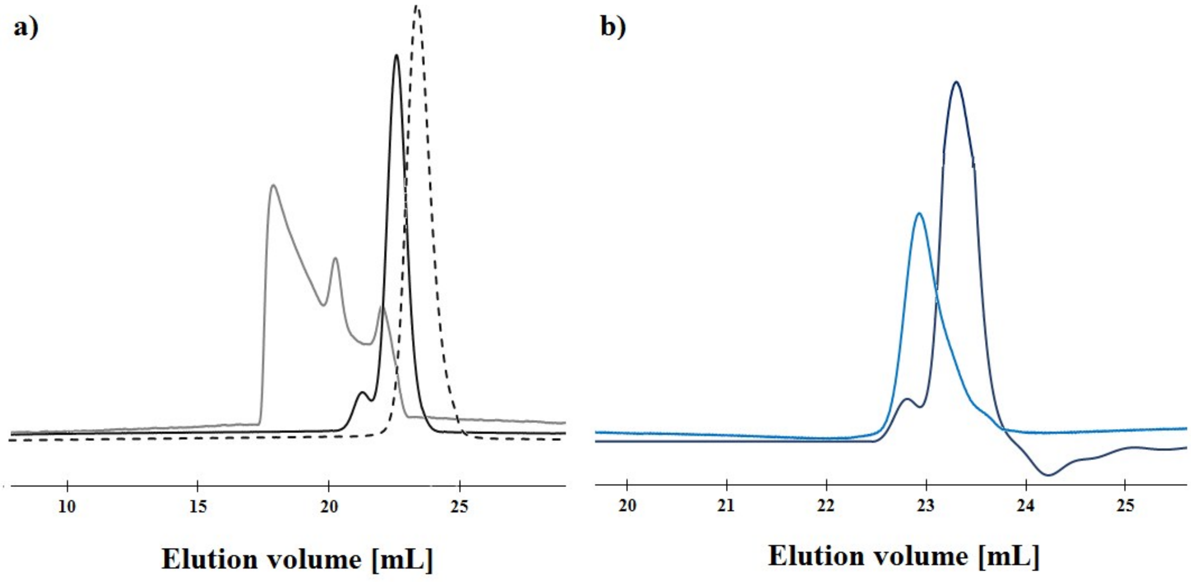

Figure 1. Panel (a): GPC-LALLS chromatograms of P[OVIM][NTf_2] produced by free-radical high pressure polymerization at p = 500 MPa (gray line), p = 800 MPa (black line) and at p = 1200 MPa (dashed line); Panel (b): GPC-LALLS chromatograms of P[OVIM][NTf_2] obtained under confinement by RAFT (blue line) and free-radical polymerization (black line).

Measurements of samples prepared by polymerization at macroscale were carried out in THF containing 10 mM $LiNTf_2$ as the solvent at 35 ^oC and a flow rate of 1 mL/min. Note that in case of GPC measurements of polymers produced under nanoconfinement, the polymer sample recovered from the AAO templates by wash with THF was first freeze-dried under vacuum, then washed with water and again freeze-dried under vacuum. Measurements were carried out in 250 μL vial inserts with a flow rate 0.9 ml/min.
